# Supplementary material for: Synovial Fluid Interleukin-16 Contributes to Osteoclast Activation and Bone Loss through the JNK/NFATc1 Signaling Cascade in Patients with Periprosthetic Joint Infection
Source: Int J Mol Sci. 2020 Apr 21;21(8):2904. doi: 10.3390/ijms21082904 (PMC7215706; doi:10.3390/ijms21082904)
Supplement: Supplementary file 1 [file ijms-21-02904-s001.pdf]

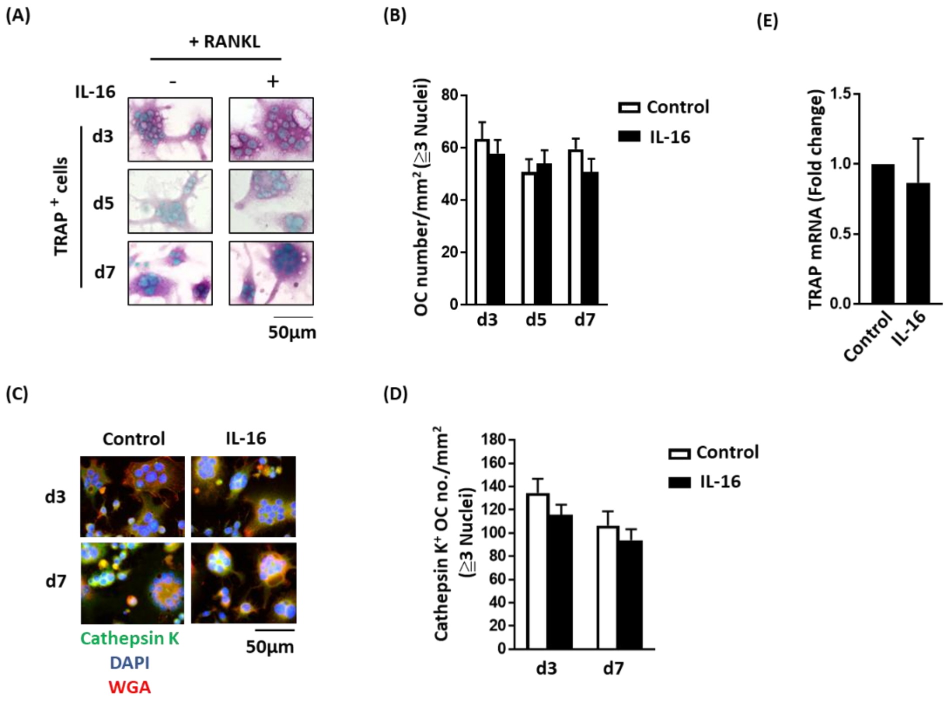


**Figure S1.** Interleukin-16 (IL-16) did not change the receptor activator of nuclear factor-kappa B ligand (RANKL)-induced differentiation of RAW264.7 cells into osteoclast-like cells. (**A**, **B**) IL-16 did not change RANKL-induced differentiation of RAW264.7 cells into tartrate-resistant acid phosphatase (TRAP)-positive osteoclast-like cells. (**C**, **D**) IL-16 did not change RANKL-induced differentiation of RAW264.7 cells into cathepsin K-positive osteoclast-like cells. (**E**) IL-16 did not enhance the RANKL-induced mRNA expression level of TRAP.

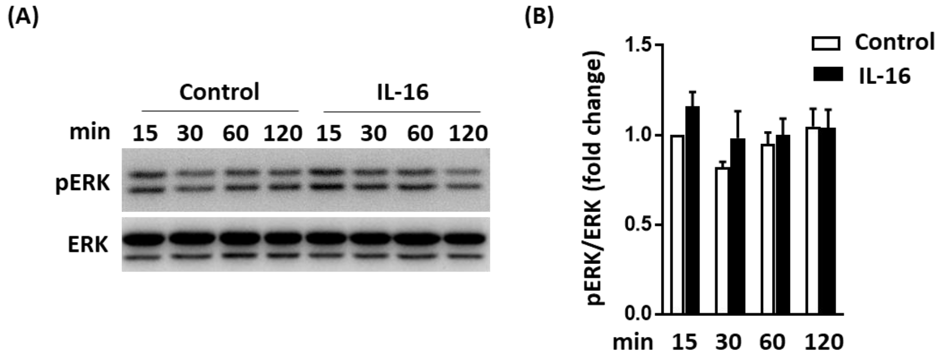


(**A**, **B**) IL-16 did not activate ERK MAPK signaling.
